# Who said dialogue conversations are easy? The communication between communication vulnerable people and health‐care professionals: A qualitative study

**DOI:** 10.1111/hex.12679

**Published:** 2018-04-19

**Authors:** Steffy E. A. Stans, Ruth J. P. Dalemans, Uta R. Roentgen, Hester W. H. Smeets, Anna J. H. M. Beurskens

**Affiliations:** ^1^ Faculty of Health Zuyd University of Applied Sciences Heerlen The Netherlands; ^2^ Department of Family Medicine CAPHRI School for Public Health and Primary Care Maastricht University Maastricht The Netherlands

**Keywords:** communication strategies, communication vulnerable people, dialogue conversations, long‐term care, patient‐provider communication, qualitative research

## Abstract

**Objective:**

To gain insight into how communication vulnerable people and health‐care professionals experience the communication in dialogue conversations, and how they adjust their conversation using augmentative and alternative communication (AAC) or other communication strategies.

**Methods:**

Communication vulnerable clients and health‐care professionals in a long‐term care institution were observed during a dialogue conversation (n = 11) and subsequently interviewed (n = 22) about their experiences with the conversation. The clients had various communication difficulties due to different underlying aetiologies, such as acquired brain injury or learning disorder. Results from the observations and interviews were analysed using conventional content analysis.

**Results:**

Seven key themes emerged regarding the experiences of clients and professionals: clients blame themselves for miscommunications; the relevance of both parties preparing the conversation; a quiet and familiar environment benefitting communication; giving clients enough time; the importance and complexity of nonverbal communication; the need to tailor communication to the client; prejudices and inexperience regarding AAC. The observations showed that some professionals had difficulties using appropriate communication strategies and all professionals relied mostly on verbal or nonverbal communication strategies.

**Conclusion:**

Professionals were aware of the importance of preparation, sufficient time, a suitable environment and considering nonverbal communication in dialogue conversations. However, they struggled with adequate use of communication strategies, such as verbal communication and AAC. There is a lack of knowledge about AAC, and professionals and clients need to be informed about the potential of AAC and how this can help them achieve equal participation in dialogue conversations in addition to other communication strategies.

## 1 INTRODUCTION

Conversations between clients and health‐care professionals are widely recognized as important because of their contribution to quality of care.^1^, ^2^, ^3^, ^4^ We define these conversations as dialogue conversations, in which essential exchanges between a client and any health‐care professional take place and in which both play a significant role. The exchanges concern, for example, health‐related goals, activity and participation choices, and evaluation of treatment. Dialogue conversations have a particularly large impact on client involvement in the health‐care process.^1^ In these conversations, effective communication is important and associated with patient satisfaction, patient safety and client‐centred care.^2^, ^5^

Effective communication can be defined as the successful joint establishment or co‐construction of meaning, using a variety of strategies, including the simultaneous use of common modalities (speech, nonverbal communication, augmentative and alternative communication (AAC)).^6^ To be truly effective, communication requires a two‐way process (expressing and understanding) in which messages are negotiated until the information is correctly understood by both parties.^7^ The present study used a broad definition of AAC, which includes formal assistive communication systems (eg voice output communication aids), conventional semiotic systems (eg handwriting), as well as commonplace objects (eg pictograms, or letters). Nonverbal communication (eg gesturing) is discussed separately.^8^

Dialogue conversations can be problematic for communication vulnerable clients, since their communication difficulties make it challenging for them to be actively involved.^9^ We define communication vulnerable people as people who experience difficulties communicating in particular situations. They struggle to express their needs, wishes and values, and/or to understand the information in conversations with professionals. This may be the result of mild to severe communication difficulties, related to their sensory, emotional, physical or cognitive abilities.^10^ Numerous underlying aetiologies and diagnoses can lead to functional communication difficulties. Acquired brain injury can lead to aphasia, dysarthria, apraxia and paralysis, which can lead to difficulties in speech and use of language. Learning disorders can lead to difficulties in understanding, memory and concentration. And physical or sensory disabilities can lead to speaking or hearing difficulties. In line with the ICF^11^ and recent developments in health care,^12^ we used a top‐down approach to examine communication vulnerability and the functional communication in conversations. This relates to the client's participation, and to the activities and participation levels defined in the ICF,^11^ and means that we focused on the experiences of clients in functional communication in conversations, rather than on the client's diagnosis (bottom‐up)^13^. It is important to acknowledge a person's experiences and elements of their environment, rather than focussing primarily on the diagnosis.^11^, ^13^

Professionals are often not aware of the clients’ communication vulnerability or do not know which strategies they can use to enable clients to express themselves or to understand the professional during dialogue conversations.^10^, ^14^ Other studies have reported that professionals can experience feelings of anxiety, fear and inadequacy when communicating with people with aphasia.^15^

However, existing studies on dialogue conversations have often focussed on a specific group of people with one specific diagnosis (eg aphasia) or do not provide in‐depth information about the functional communication problems that both professionals and clients experience.^16^, ^17^, ^18^ It is important to address the broad target group of communication vulnerable people, regardless of their underlying diagnosis or symptoms,^19^ to be able to focus on ways of adapting communication to the specific needs of an individual client.^20^ Furthermore, research about communication in clinical practice is mostly targeted at the process steps of dialogue conversations^21^ or affective factors such as trust, respect and empathy, missing a focus on communication and AAC.^22^, ^23^ There is a lack of knowledge about the communication experiences of both communication vulnerable people and professionals, especially with regard to the way they overcome communication problems and use communication strategies during dialogue conversations.^5^, ^10^ Research into the communication experiences of communication vulnerable people is challenging, due to their communication difficulties.^10^, ^24^ Although quantitative data can be used, this does not provide in‐depth information about the way they experience their communication during dialogue conversations. Such insights are needed to advise professionals on how to engage with communication vulnerable clients.

Therefore, the purpose of this study was to gain insight into how communication vulnerable people and health‐care professionals experience the communication in dialogue conversations, and how they adjust their conversations using AAC or other communication strategies.

## 2 METHODS

A qualitative study was conducted, based on general tenets of naturalistic inquiry, focussing on communication in the natural setting of a care institution.^25^ Observations were followed by semi‐structured interviews with both clients and professionals.

### 2.1 Setting and participants

This study was conducted in a long‐term care institution for people with acquired brain injury and physical limitations in the Netherlands. The local client advisory board advised the researchers about selected sites where they could find clients with a variety of communication difficulties who required various types of support (eg medical, living, daily activities). Professionals who regularly had dialogue conversations with clients were recruited by the managers using convenience sampling. Clients were recruited by the selected professionals using purposive sampling based on the following selection criteria: being older than 18, not completely blind or deaf, able to communicate experiences (with or without AAC), having at least one dialogue conversation every 6 months with the professional, and providing more than two “yes” answers on the communication vulnerability screening list (Appendix S1).

### 2.2 Data collection

Between March and July 2015, two researchers (SS, HS) observed dialogue conversations between pairs of professionals and clients. Immediately afterwards, the client was interviewed first (to prevent problems of recalling the conversation), followed by the professional. Each interview was conducted by two trained interviewers (SS, HS) using a self‐developed interview guide that focussed on experiences of communication, adaptations and AAC. The questions were formulated using the literature about communication and AAC,^5^, ^6^, ^10^ supplemented by several additional items that emerged during the observations. The interview guide was discussed with the local client advisory board to enhance its accessibility. Different types of questions were tailored to the abilities of the clients, with or without pictograms showing several answer options, using short sentences and high‐frequency words and providing sufficient time and short breaks.^24^ In addition, probing questions were used and the researchers took care to note nonverbal behaviour that indicated understanding of the questions. Field notes were taken after each observation and interview.

### 2.3 Data analysis

The interviews and observations were audiotaped, transcribed verbatim and analysed using conventional content analyses.^26^ Two researchers (SS, HS) read the transcripts repeatedly and assigned codes to relevant fragments using the qualitative analysis software NVivo 11. Coding was derived directly from the text, focussing on experiences, adapting communication to the clients and the use of AAC. During their discussions, overarching themes emerged from the data, and the codes and themes were constantly compared between the observations, field notes and interviews. Other researchers (RD, AB, UR) took part in peer debriefing sessions where they reflected on the analysis.^25^ The themes were adjusted until a final thematic structure was decided on by all researchers. After 20 interviews and 10 observations, no new themes emerged and therefore we assumed that thematic saturation had been attained; the final two interviews served to confirm and verify the content analysis.

To ensure internal validity, the preliminary analysis of the first three interviews was discussed with the client advisory board as an intermediate member check. After full analysis, another member check was performed by sending the participants a summary of the thematic results in accessible form.^24^

### 2.4 Ethical considerations

The local Human Research Ethics Board Z (Heerlen, The Netherlands), which verifies if studies are conducted in accordance with the Declaration of Helsinki^27^ and other appropriate EU regulations and laws, approved this study. Those willing to participate first provided verbal consent to the professional who had recruited them, and additionally written or audiotaped informed consent to the researchers in accessible format.^24^

## 3 RESULTS

In total, 11 observations and 22 interviews were conducted. The clients represented a heterogeneous group with considerably different scores on the communication vulnerability screening list (see Table 1). At the time of the study, none of the clients was consulting a speech and language pathologist, and only one of the clients occasionally used an AAC, namely a picto‐book. The aim of the dialogue conversations differed, ranging from issues such as goal setting to the client's satisfaction with the care process. The median duration of the conversations was 14 minutes (range 5‐47).

**Table 1:** Characteristics of the participating clients and professionals (using fictitious names)

Client	Age	Gender	Diagnoses of clients: aetiology	Positive scores on screening list	Professional	Age	Gender	Occupation	Years of working in setting	Setting
Mark	40	♂	ABI	1, 3, 6‐12, 14, 16, 18,19, 20, 22‐24 (n = 17)	Huub	62	♂	DAS	30	AC
Hendrik	75	♂	NDD	1, 6, 8‐11, 14, 17‐20, 24 (n = 12)	Maria	64	♀	DAS	21	AC
Peter	60	♂	ABI	1, 2, 6, 8‐12, 14, 16‐19 (n = 13)	Karin	26	♀	DAS	5	AC
Erik	55	♂	ABI SCI	1, 2, 7, 17, 24 (n = 5)	Mandy	32	♀	DAS	5	SL
Linda	27	♀	CP	1, 2, 4, 6, 8, 9, 14, 17 (n = 8)	Anne	48	♀	N	26	SL
Johanna	57	♀	MS	2, 7, 13, 17, 21(n = 5)	Monique	52	♀	N	18	SL
Edwin	40	♂	ABI, CC	1‐4, 5,6, 9, 21, 23, 24 (n = 10)	Bart	42	♂	SPN	12	SL
Astrid	57	♀	Stroke	1‐14, 17 (n = 15)	Vera	50	♀	N	31	SL
Jan	55	♂	Vanishing white disease	1, 2, 4, 9‐12, 14, 19, 23, 24 (n = 11)	Daisy	57	♀	DAS	38	SL
Kevin	25	♂	Duchenne muscular dystrophy and learning disorder	6, 7, 9, 18, 19, 23, 24 (n = 7)	Sandra	52	♀	N	20	SL
Esther	40	♀	CP, MS	1, 2, 4‐9, 12, 18, 20 (n = 11)	Laura	38		DAS, OT	16	AC

ABI, acquired brain injury; AC, activity centre; CC, Contusio Cerebri; CP, Cerebral Palsy; DA, daily activity supervisor; MS, Multiple Sclerosis; N, nurse; NDD, Neurodegenerative Disease; OT, occupational therapist; SCI, Spinal Cord Injury; SL, supported living facility; SPN, social psychiatric nurse.

**Figure 1:**
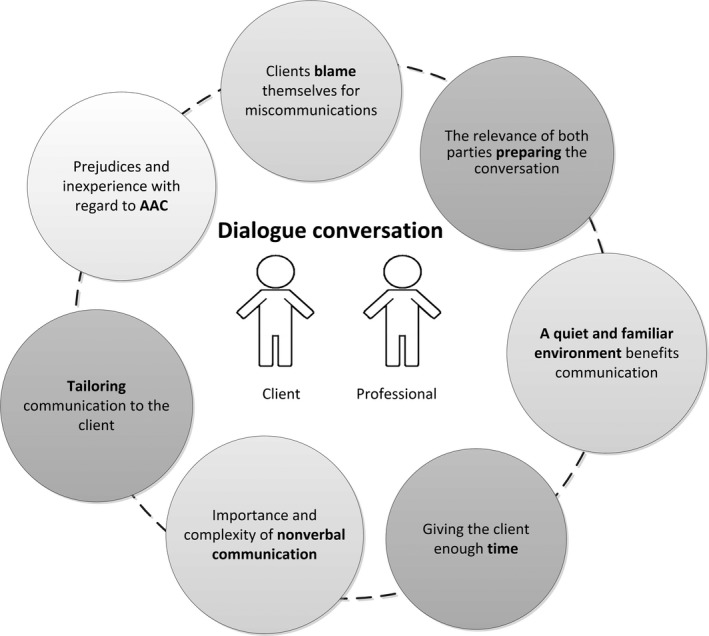
Themes relating to the experiences regarding the dialogue conversations, and the adjustments made

The content analysis revealed seven key themes (Figure 1). The results of the interviews and observations reinforced each other and are therefore presented together in the results section. Within each theme, we describe the perspectives of clients and professionals, as well as our insights from the observations.

### 3.1 Clients blame themselves for miscommunications

The clients tended to take responsibility for communication problems during the conversations: they blamed their own disability. They explained that they could not understand difficult words because of their cognitive problems or that the professional did not understand them because of their speech problems.

Interviewer “Yes, and she did not understand it?”

Peter (client) “No!”

Interviewer “OK, and why didn't she understand it?”

Peter (client) Murmurs and points to himself.

The professionals did not mention this topic explicitly, but they did describe a need for adapting their communication to the client's disabilities. The question of blame was not discussed during the observed conversations.

### 3.2 The relevance of both parties preparing the conversation

Both clients and professionals found it important to prepare the conversation and found it helpful to receive written information prior to the conversation. Several clients mentioned that this gave them time to think about the subject.

Some professionals prepared the conversation by preparing a fixed structure of topics to discuss. Others described supporting the clients by asking them to think about what they wanted to discuss.

Anne (professional) “If she is very tense then nothing comes out, but if she has a reminder on paper, then she thinks “Oh right, that's what I wanted to talk about”; for her, that's a kind of preparation.”

The observations showed that most professionals prepared the conversation, but only for themselves. The clients were not always informed about the structure or content in advance and often seemed to follow the professionals’ lead. For example, in conversation 2 the professional had brought along a list of goals to evaluate, which she used as a support for herself; the client had not received this information. However, in observation 9, the structure prepared by the professional was appropriately tailored to the client who had memory problems and he could follow the structure.

### 3.3 A quiet and familiar environment benefits communication

The clients and professionals expressed that a calm and quiet environment without distractions is important in conversations. Noise makes it difficult for clients to concentrate or remember what the conversation is about. Background noise also hampered the professionals’ ability to understand their clients. The clients specifically mentioned that it helped them to express themselves if the conversation took place in an environment where they felt comfortable, for example in their own living environment.

Interviewer “Do you always have the conversations here [ie his own apartment]?”

Kevin (client) “Yes, I feel comfortable here, it's more comfortable, and quiet, right?”

The researchers also observed that the conversations in the supported living facilities mostly took place in the client's own room, which was a quiet environment, with the door closed, and no other people present.

### 3.4 Giving the client enough time

The clients found the professionals’ time investment and patience very important, because they often need a lot of time to express what they wanted to convey, to complete their sentences or to come up with words.

Linda (client) “Let me complete my sentences, don't do the talking for me, let me talk.”

Some clients had had unfavourable experiences, feeling that there was not enough time available for them to express themselves, or that the professionals completed their sentences for them.

Interviewer “How could she have helped you to make that clear?”

Peter (client) “Yes but erm…it makes…waiting…but that erm…”

Interviewer “ (…). And do you have the feeling she took enough time to discuss that with you?”

Peter “No.”

Others had had favourable experiences, for example when the professional showed patience while the client stuttered.

A few professionals also emphasized the importance of giving clients enough time, time to stutter, to find their words, or to process the information.

Monique (professional) “We must be careful, because you've worked here for so long, you know a lot about clients, that you do not quickly erm, provide the answer yourself (…) then you tend to, if they say one letter, to fill the rest in for them.”

The researchers observed that time was not always used efficiently. Conversations that took longer did not necessarily mean that clients had more time to express themselves. For example, while conversation 2 took 28 minutes, the professional talked fast, used long sentences, completed the client's sentences and asked multiple questions at a time. These actions meant that less in‐depth information was received from the client. By contrast, the researchers observed that in conversation 5, which took only 9 minutes, the client who stuttered was encouraged to complete her own sentences and to initiate topic shifts.

### 3.5 Importance and complexity of nonverbal communication

The clients stated that nonverbal communication was very important for them to express themselves, for example using gestures in combination with speech.

Interviewer “Do you use any aids to help you communicate, talk?”

Peter “Yes (makes a lot of gestures).”

Interviewer “Gestures?”

Peter “Yes.” (keeps making gestures).

The professionals also reported that nonverbal communication, specifically facial expressions, body language and eye contact, was important to understand the client better or to ascertain whether the client understood them.

However, the professionals also explained that the nonverbal communication of communication vulnerable clients was complex and often difficult to interpret, due to physical disabilities such as spasms. Knowing the client well helped them interpret the nonverbal communication.

Vera (professional) “At a specific moment you just notice, (…) for example that she keeps adjusting the seating position of her electric wheelchair, she cannot sit still any more, yes then the conversation is taking too long.”

The researchers observed that clients used a lot of nonverbal communication, mainly gestures, and the professionals did pay attention to this. In fact, some professionals relied almost entirely on nonverbal communication. The client and professional in conversation 1 had a conversation relying only on nonverbal signs and the client's yes/no/hmm answers. This, however, restricted the client in introducing a topic, feeling, or thought of his own.

Huub (professional) “I think I already see in your eyes what you want to do?”

### 3.6 Tailoring communication to the client

The next three subthemes describe the experiences of clients and professionals as regards tailoring the communication.

#### 3.6.1 Tailoring communication speed and complexity

The clients described that the professionals had helped them to better understand the conversation, by repeating information and speaking slowly. However, some of the clients could not always understand the professionals, because they used difficult words, talked too fast, used sentences that were too long, or gave too much information.

Interviewer “Not quite, okay, …what didn't you understand?”

Kevin (client) “The difficult words.”

Professional 5 explained that using simple language helped the client to understand her. The professionals emphasized the importance of adapting the conversation to the clients’ degree of tiredness and their concentration, mood and cognitive abilities.

The researchers observed that in conversations 1 and 2 the professionals talked fast and used long sentences, they asked for clarification many times (13 and 14 times), indicating difficulties in understanding each other (Table 2), and clients had difficulties following the conversation or responding to questions. In other conversations, the professionals talked calmly and clearly and enhanced understanding using examples.

**Table 2:** Results of transcribed observations

No.	Duration	Topic chosen by	No. of questions asked		No. of times topic shifted		Completing a sentence of the conversation partner		Interruption for confirmation		Interruption		No. of times clarification was asked	
			By client	By prof.	By client	By prof.	By client	By prof.	By client	By prof.	By client	By prof.	By client	By prof.
1	14 min	Together	0	142	0	12	0	3	6	3	4	4	0	13
2	28 min	Professional	2	104	2	4	3	13	45	29	14	24	0	14
3	5 min	Professional	1	19	2	8	0	2	8	2	2	2	1	2
4	32 min	Together	6	8	4	9	1	3	6	7	7	10	3	3
5	9 min	Client	0	29	13	1	1	4	14	12	4	4	0	9
6	9 min	Client	0	6	7	2	0	1	8	20	7	4	0	1
7	40 min	Together	5	28	11	7	1	1	54	59	22	23	1	4
8	8 min	Client	0	25	4	2	0	2	21	9	3	2	0	4
9	15 min	Professional	0	46	9	13	1	2	24	11	5	4	0	2
10	10 min	Together	1	27	1	5	1	1	32	1	5	3	0	3
11	47 min	Together	11	137	10	4	4	1	38	26	36	12	3	14

min, minutes; no., number; prof, professional.

#### 3.6.2 Preparing a structure for both professional and client

The professionals emphasized the importance of structuring the conversation, using a predefined structure, summarizing, paraphrasing and guiding the client back to the topic of conversation. The clients did not mention the concept of structure.

The observations showed that some clients had difficulties staying on topic and following the conversation. In conversations 4, 6, 7 and 9, the professionals managed to guide the clients back to the topic while also giving them enough time to tell their story. Their strategies involved: paraphrasing, asking questions, clearly indicating a topic shift and pointing it out to them when they deviated from the topic.

Bart (professional) “Of course that has to do with his brain injury (…), then you have to get him back to the subject we were talking about (…) I first let him talk, then I say “okay fine, but let's go back to the topic we were talking about”.”

#### 3.6.3 Tailoring questions to the client's needs

The professionals described that it helps the clients to ask one question at a time; the clients did not mention this strategy. However, the observations showed that not all professionals used this strategy. In conversations 2 and 3, the professionals asked multiple questions at a time, leading to unclear answers from the clients.

Karin (professional) “But, do you think, like, I need to keep working on this goal? Or do you say, now I'm ready? Now it's ready, now I don't need to work on it.”

Hendrik (client) “Yes that's right.”

The professionals used both open and closed questions and reported that using closed questions could help the clients. However, the observations showed that using too many closed questions led to a lack of depth in conversations 1, 2 and 3 and that in these situations clients struggled to initiate a topic shift. During conversation 2, the professional initiated 12 topic shifts and the client none (Table 2). In contrast, other observations showed that professionals who used mostly open questions and follow‐up questions supported the client in initiating topic shifts. Observation 5 shows that the professional asked 28 questions, the client none, but the open questions enabled the client to introduce 13 topic shifts (Table 2).

### 3.7 Prejudices and inexperience with regard to AAC

The clients did not know if they would like to use AAC, due to a lack of experience. Some clients thought pictograms were childish, while others found them helpful during the interview with the researchers.

Interviewer “Do these pictograms help you?”

Mark (client) “Yes!”

A few clients explained that it is helpful to use conventional semiotic systems, such as writing, to express themselves during a conversation.

Interviewer “What could she have done to enable you to tell it? Except for giving more time.”

Peter (client) “Plants, pen.”

Interviewer “Pen? Oh, she could have written it down? (client shakes his head) Oh, she could have given you the pen?”

Interviewer gives Peter paper to write on, Peter writes down “plant” to indicate which topic he wanted to discuss.

The professionals explained that they did not use formal assistive communication devices or pictograms because they thought it was not necessary, it was childish, or it was for “stupid” or “crazy people”.

Interviewer “And do you ever use communication devices to talk to him?”

Sandra (professional) “Yes, he uses the clock he has with pictograms, but apart from that no, that is totally not necessary’(…) ‘That's because he's not stupid right, it's more like yeah he's not stupid.”

Two of the professionals indicated that clients could benefit from photos or a picto‐book, but did not use this strategy during the observed conversations.

Other professionals described that written information would probably help the client to understand them, or to remember what was said. Such written information had to be adapted to the client's abilities, presenting it in large font, including only a limited amount of information, and using simple words.

Anne (professional) “Then I write it down on paper in advance, using a larger font, so that she can read it more easily (…) No difficult words and not too much information.”

The observations showed that only one professional, Laura, used written information, by using the computer. Client and professional described that it was helpful for the client to hear as well as read the information.

Even though the use of formal assistive communication systems, pictograms, written information and writing were sometimes mentioned as helpful, the researchers observed that these were not used in the dialogue conversations.

## 4 DISCUSSION

The purpose of this study was to gain insight into how communication vulnerable people and health‐care professionals experience the communication in dialogue conversations, and how they adapt their conversations to their clients using AAC or other communication strategies. Seven key themes emerged: the question of blame, preparing the conversation, the environment of the conversation, giving clients enough time, nonverbal communication, tailoring communication and prejudices regarding AAC. Clients and professionals acknowledged the wide range of communication strategies, but our observations showed that they mostly relied on verbal and nonverbal communication, and did not use AAC. Clients were often insufficiently enabled to express themselves, whereas client‐centred care and shared decision‐making require an active role of clients in dialogue conversations.^28^

It is striking that the clients thought they were to blame for difficulties in the conversation. Clients were not aware that professionals could have used AAC to enable them to become more involved.

Sufficient time was considered important by clients and professionals; however, more time was not always the solution. The duration of the conversations we observed fluctuated, regardless of where they took place, that is at an activity centre or assisted living facility. Time must be used efficiently, using the right communication strategies tailored to the client, supporting effective communication and involving clients in their health‐care process.^1^, ^2^, ^19^

Professionals and clients agreed about the importance of preparing conversations and ensuring a suitable environment, which has also been emphasized in previous research.^29^ Our observations showed that environmental issues were taken into account, but the preparation mostly did not include the clients.

The current study showed that the professionals had difficulties using adequate communication strategies. Whenever clients found it difficult to talk and remained silent, some professionals filled the silence with information or questions. This finding is in agreement with those reported by Wylie and colleagues, who stressed that communication vulnerable people often do not receive the support they need to overcome their communication difficulties.^14^ The three conversations in which the professionals and clients had the most difficulties in their conversations all took place in an activity centre. However, we cannot link the lack of skills and awareness shown by the professionals to the type of facility, since this study had a limited number of participants.

This study showed that asking more questions appeared to be not necessarily better or worse, but that the types of question need to be tailored to the client and his or her communication difficulties. The study by Gordon and colleagues, including people with aphasia and dysarthria, also found that nurses often controlled the topic of the conversation, while clients were limited to responding to closed questions.^16^ Another study found that clients are often not enabled to initiate a new topic or provide new information.^30^ It could be concluded that awareness of communication vulnerability^10^, ^14^ and awareness regarding effective use of communication strategies are both needed in order to enable clients to be more involved.

In the current study, the professionals and clients did not use formal assistive communication devices such as picto‐books, dynamic communication devices or conventional semiotic AAC, such as writing, drawing or photographs, which are readily available. This is remarkable considering the communication vulnerable group. This lack of AAC use was noticed both in activity centres and in assisted living facilities. Blackstone and colleagues also identified a lack of AAC use in hospitals.^31^ It is a striking result, since AAC could support clients in expressing their views and preferences,^32^, ^33^ providing them with the opportunity to be more fully engaged in the conversation. Some professionals in the current study also had negative prejudices about AAC, which indicates some sort of stigma on using AAC.^34^, ^35^ Therefore, it is important to pay attention not only to diagnostic factors but also to environmental and psychosocial factors when choosing communication strategies. Some professionals in this study were aware of the client's diagnosis, but did not always understand the relation between the diagnosis and the difficulties emerging in the communication, indicating a lack of knowledge about communication disability. This is in accordance with previous studies, which indicated that professionals are insufficiently aware of the communication vulnerability and the potential of AAC.^10^ AAC is frequently seen as a last resort, while professionals can involve the clients in choosing and using AAC from the moment a difficulty in communicating arises.^33^

## 5 STRENGTHS AND LIMITATIONS

In our qualitative design, data triangulation was ensured by combining field notes, observations and interviews. The heterogeneous sample of clients with various communication difficulties, and the description of the contexts (thick description)^25^ may imply transferability of the thematic results. However, professionals should take cultural differences and the relatively small sample of this study into account.

Including communication vulnerable people in this study required significant time and effort, but provided much added value. Many studies about communication do not include the views of the clients or the users of AAC, whereas this vulnerable group in particular needs to have a voice and be heard.^4^, ^14^, ^36^

Weaknesses of this study relate to potential bias due to sampling and socially acceptable answers.^25^ The professionals could have chosen the clients who they thought were satisfied with the conversations, and the managers could have chosen professionals who they thought had good or poor communication skills. We used a preliminary member check with clients, but did not include the views of professionals. Furthermore, we cannot be sure that no socially acceptable answers were given during the interviews. The information letter and informed consent, however, clearly indicated confidentiality. The qualitative nature of this study and the heterogeneity of the participants prohibit the investigation of interrelations between certain characteristics of participants (eg diagnosis), setting (eg activity centre or supported living) and the functional communication difficulties experienced. Future research with a more homogeneous target group and a larger sample could provide insights into the association between functional communication difficulties experienced, diagnosis and effective communication strategies.

## 6 CONCLUSION

Both clients and professionals appreciated the benefits of preparing the conversation, ensuring a suitable environment for the conversation, giving clients enough time, using nonverbal communication and tailoring communication to the clients. However, appropriate application appears to be complex and difficult. Our findings show that these conversations are skewed towards the professionals, their preparation, their structure, their topics and their opinions about AAC. There is room for improvement since clients are often insufficiently supported in expressing themselves or understanding the professional, thereby limiting their involvement in the conversation. Professionals could use the screening list we developed to identify hidden communication difficulties. This study highlights that professionals are often unaware that using AAC can empower clients to be more involved in conversations. Future research should examine how professionals and clients can select and use communication strategies, including AAC, to help them achieve equal participation in dialogue conversations. Studies about the link between the functional communication experienced and the communication strategies/AAC tools, and about shared decision making, would be particularly interesting for dialogue conversations.

## CONFLICTS OF INTEREST

No conflicts of interest have been declared.
