# Supplementary material for: Who said dialogue conversations are easy? The communication between communication vulnerable people and health‐care professionals: A qualitative study
Source: Health Expect. 2018 Apr 19;21(5):848–57. doi: 10.1111/hex.12679 (PMC6186534; doi:10.1111/hex.12679)
Supplement: Supplementary file 1 [file HEX-21-848-s001.docx]

**Appendix 1 Screening List Communication Vulnerability**

Instruction: Please answer all questions on this page by crossing the boxes per question.

| **Question** | **Yes** | **No** |
| --- | --- | --- |
| 1. Does the individual have a diagnosis (e.g., autism, CP, aphasia, ALS, etc.) that puts him/her at risk for speech and language challenges? |  |  |
| 1. Does the person have difficulties with attention during the conversation? |  |  |
| 1. Does the person have difficulties with vision during a conversation? |  |  |
| 1. Does the person have difficulties with memory during a conversation? |  |  |
| 1. Does the person have difficulties with turn taking during a conversation? |  |  |
| 1. Does the person have difficulties with understanding speaking partners? |  |  |
| 1. Does the emotional condition of the person impact conversations negatively? |  |  |
| 1. Does the person have physical limitations that impact having a conversation? |  |  |
| 1. Does the person have difficulties with processing information during a conversation? |  |  |
|  |  |  |
| 1. Does the person use speech, but the speech is unintelligible? |  |  |
| 1. Does the person have less than 20 words or signs/signals that can be understood by familiar listeners? |  |  |
| 1. Does the person have difficulties with expressing his/her basic needs? |  |  |
| 1. Does the person have difficulties with expressing a clear yes or no? |  |  |
| 1. Does the person attempt to communicate verbally, but attempts are unintelligible to most listeners (due to for example apraxia or aphasia)? |  |  |
| 1. Does the person have difficulties with understanding nonverbal communication of others (e.g., facial expressions, gestures)? |  |  |
| 1. Does the person have difficulties with expressing nonverbal communication (e.g., facial expressions, gestures)? |  |  |
|  |  |  |
| 1. Does the person become frustrated (e.g., giving up, angry) because he/she has difficulties with communicating with others? |  |  |
| 1. Is it difficult for the person to successfully participate in meaningful day-to-day activities (e.g., leisure, work, school) due to communication difficulties? |  |  |
| 1. Does the person show an interest in social interaction, but lacks the verbal skills to do so? |  |  |
| 1. Does the person have difficulty initiating interaction with others? |  |  |
|  |  |  |
| 1. Is there a lack of using objects, photographs or picture symbols to express him/herself? |  |  |
| 1. Does the person use body language or gestures that others do not understand? |  |  |
| 1. Do visual aids increase understanding and expression of the person? |  |  |
| 1. Does the person have less verbal skills in comparison with peers? |  |  |

**Number of yes answers:….**

Remarks:

……………………………………………………………………………………………………………………………………………………………

……………………………………………………………………………………………………………………………………………………………

……………………………………………………………………………………………………………………………………………………………

……………………………………………………………………………………………………………………………………………………………

*This screening list is developed by Zuyd University of applied sciences, research center of Autonomy and Participation of people with a chronic illness. The list is based upon the ‘Communication Success Screening’ of Dynavox Mayer-Johnson; the screening list ‘starten met ondersteunde communicatie?’ by Modem; and the developmental model of ‘Taal Centraal’ (2009) of prof. van Balkom.*
